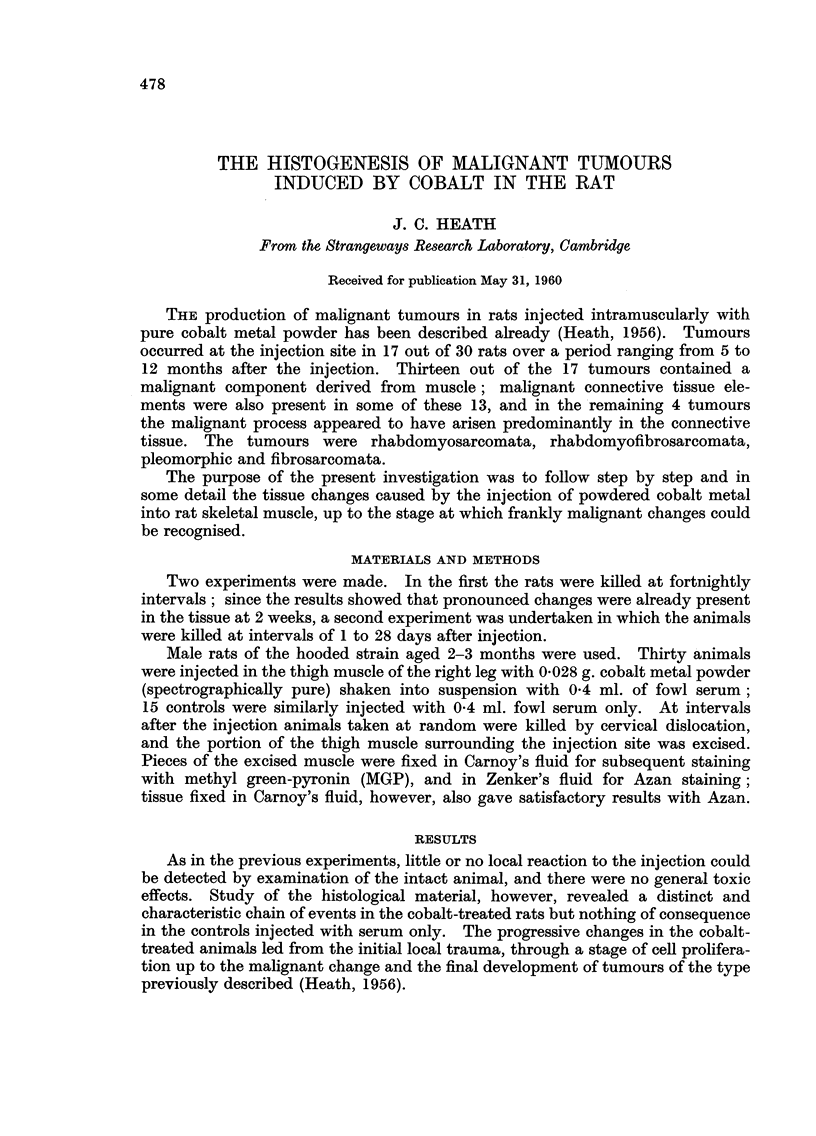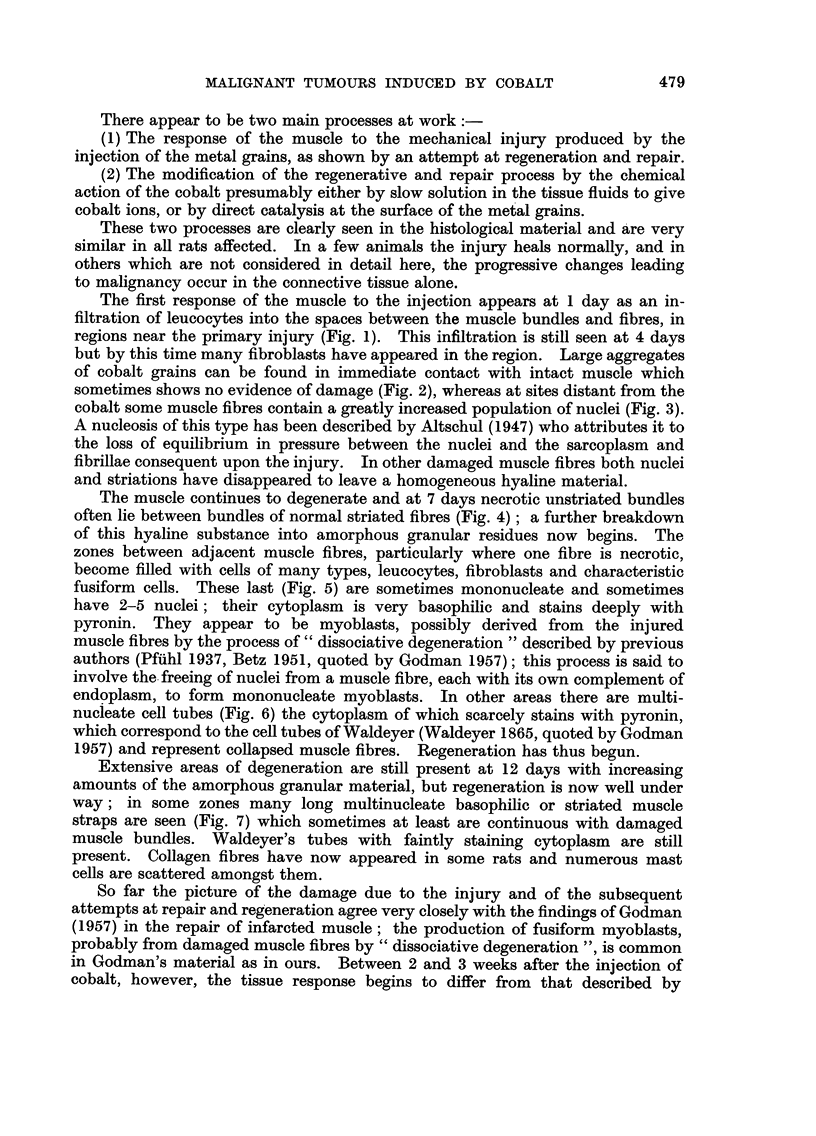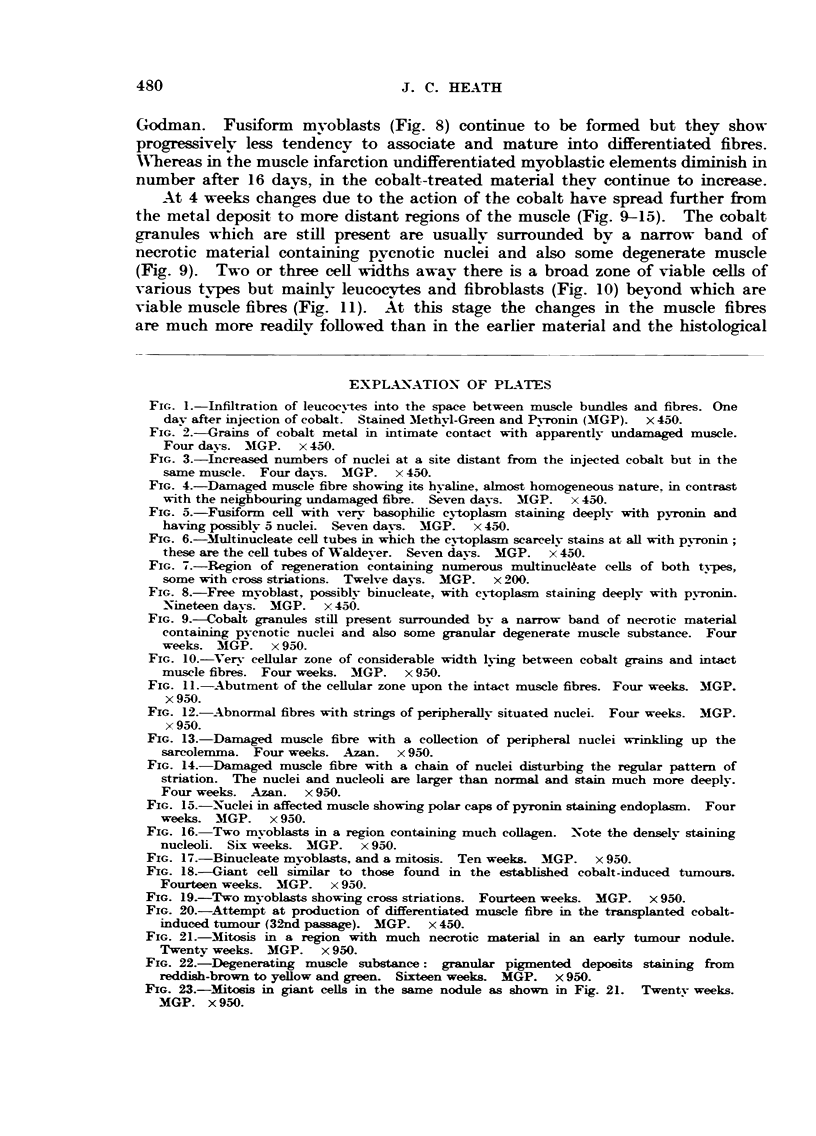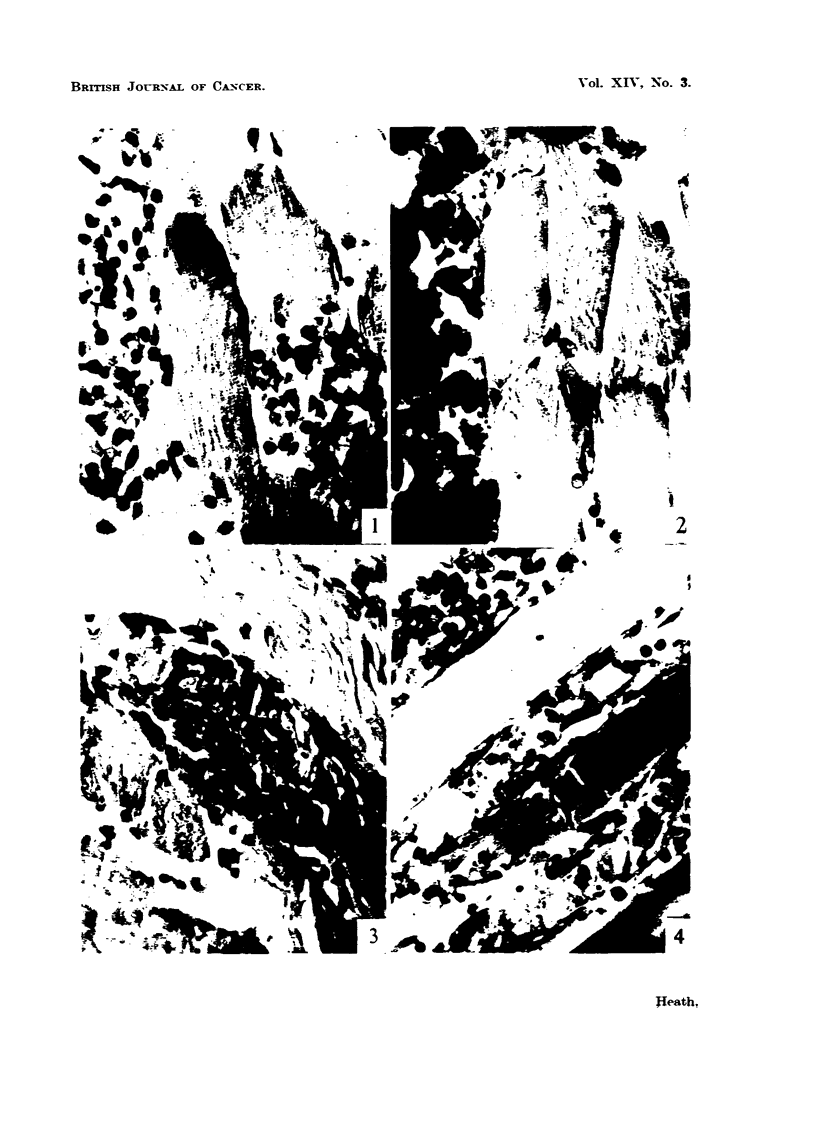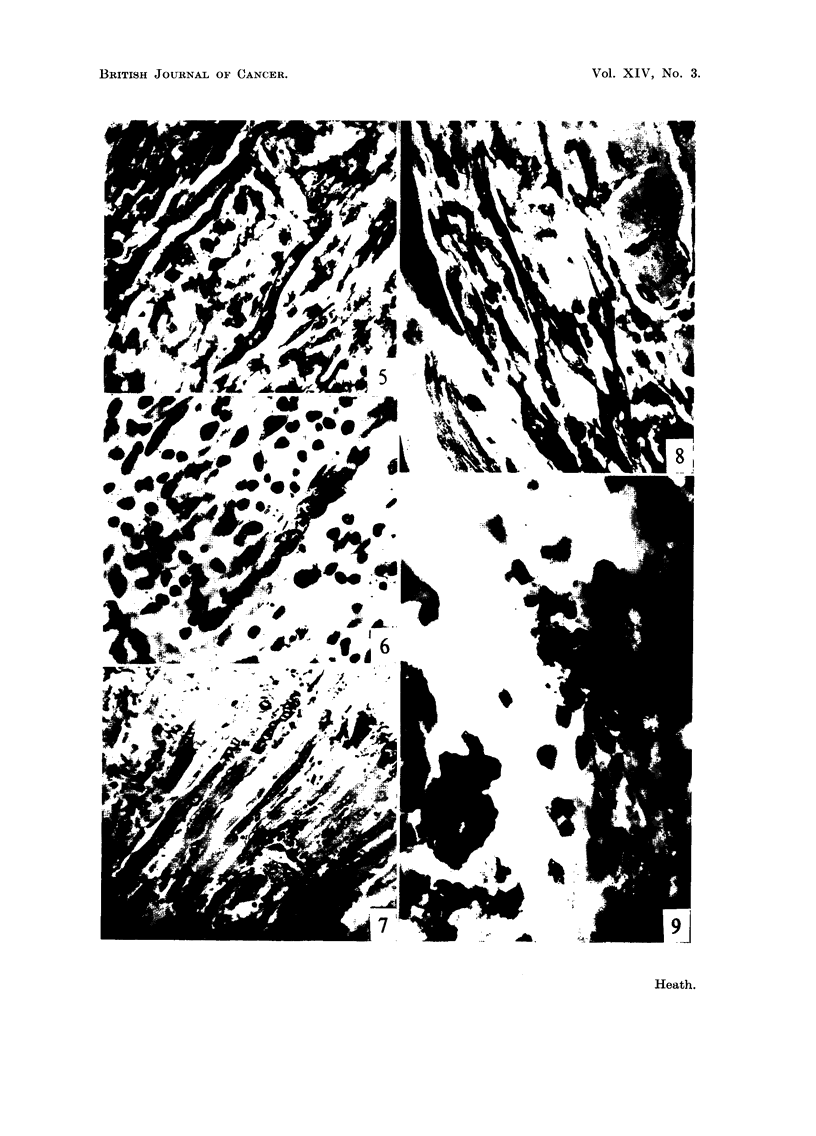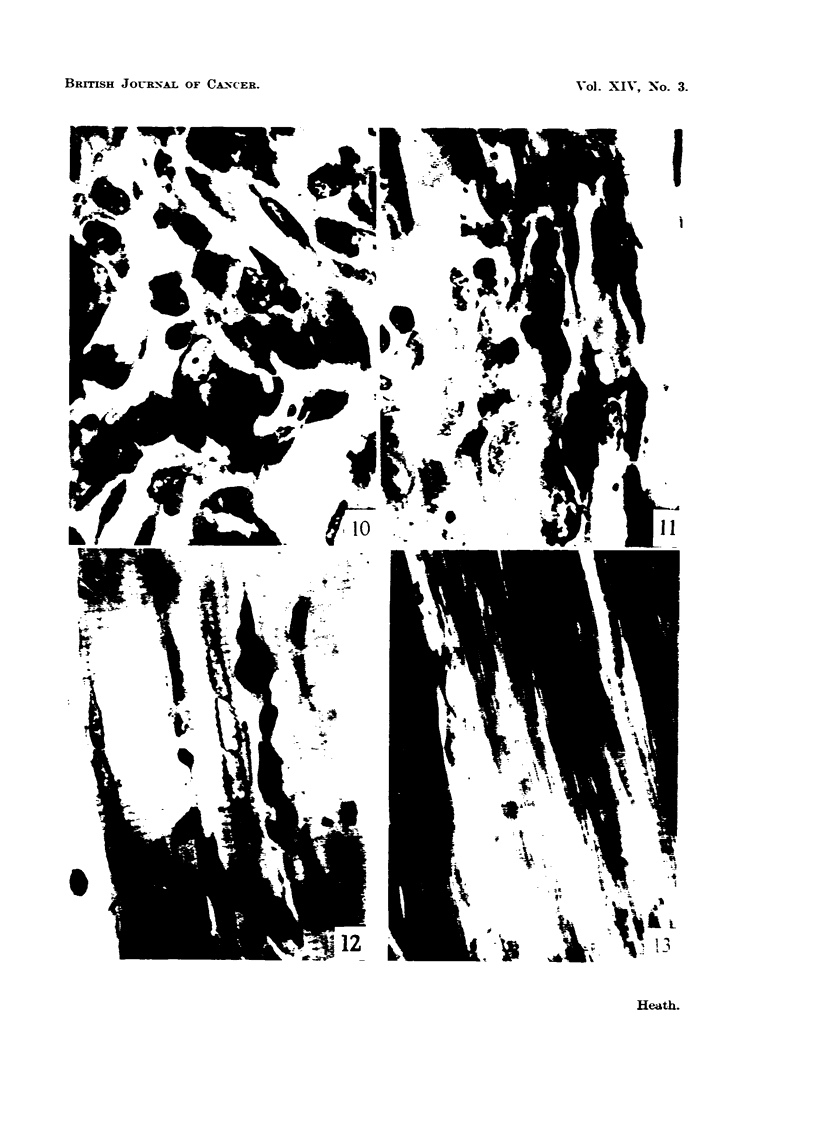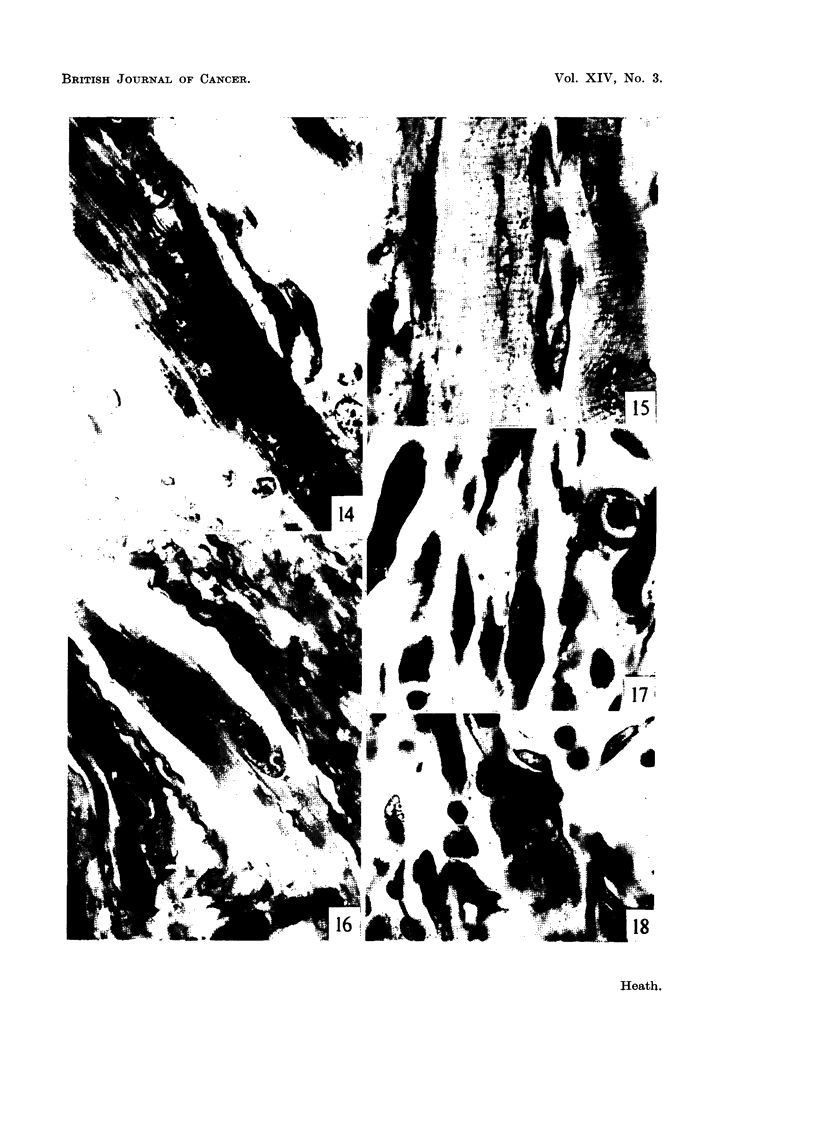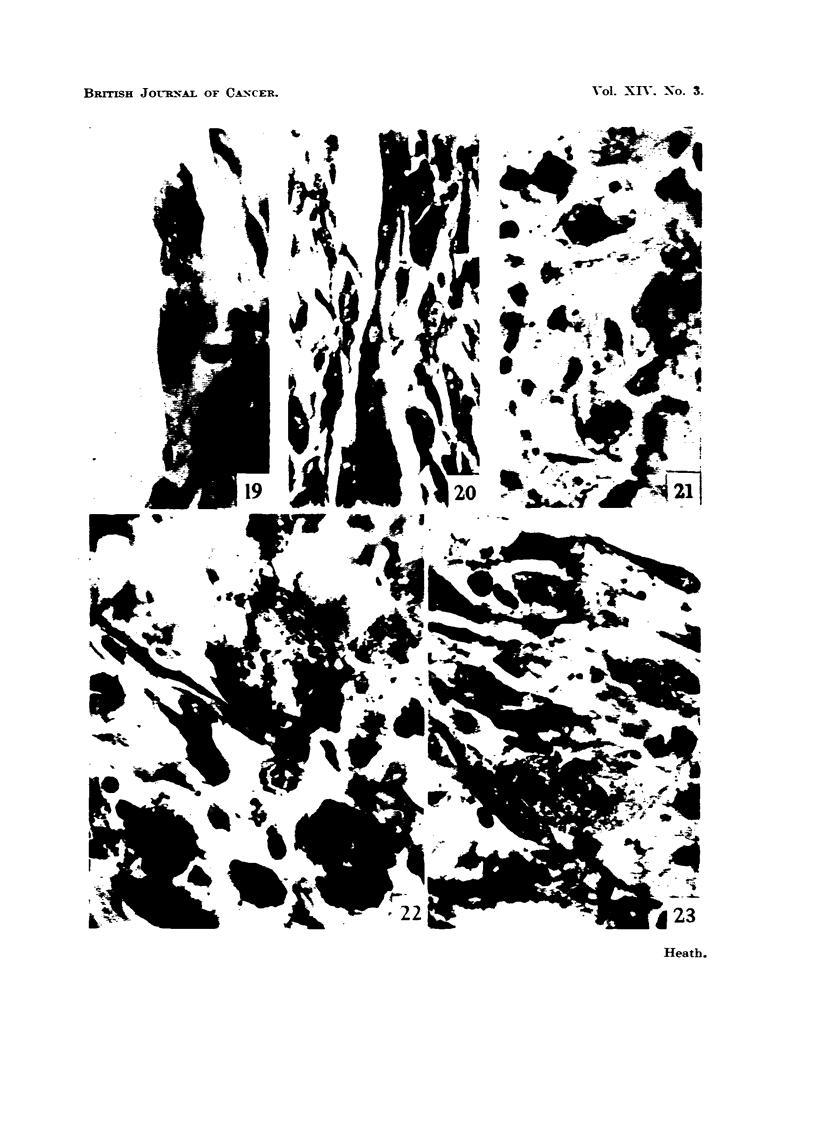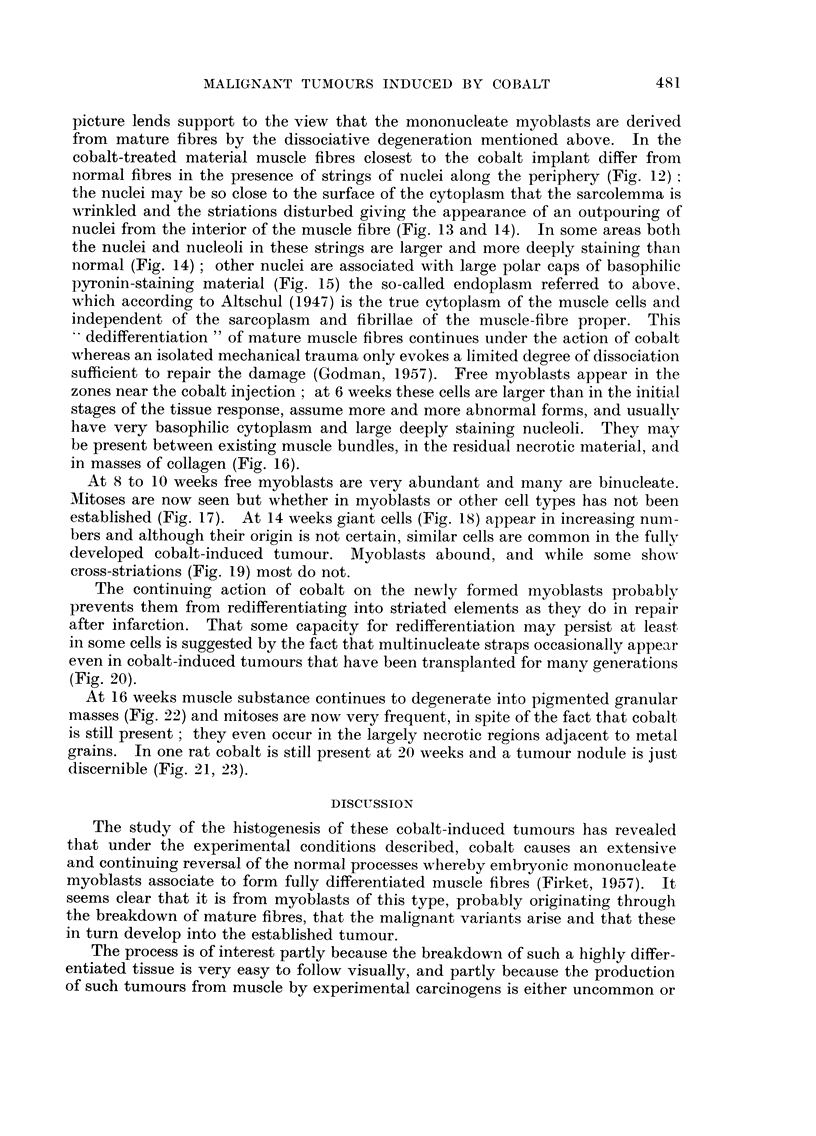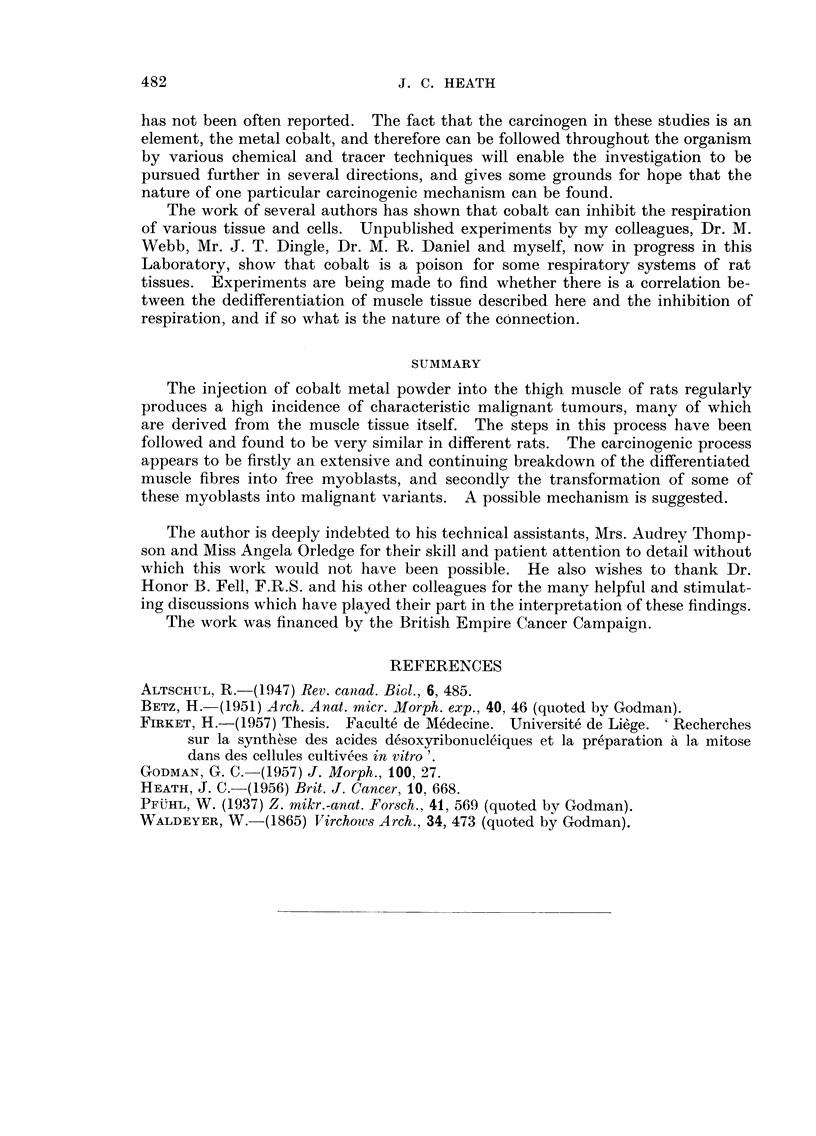# The Histogenesis of Malignant Tumours Induced by Cobalt in the Rat

**DOI:** 10.1038/bjc.1960.52

**Published:** 1960-09

**Authors:** J. C. Heath

## Abstract

**Images:**


### THE HISTOGENESIS OF MALIGNANT TUMOURS INDUCED BY COBALT IN THE RAT

J. C. HEATH

*From the Strangeways Research Laboratory, Cambridge*

Received for publication May 31, 1960

THE production of malignant tumours in rats injected intramuscularly with pure cobalt metal powder has been described already (Heath, 1956). Tumours occurred at the injection site in 17 out of 30 rats over a period ranging from 5 to 12 months after the injection. Thirteen out of the 17 tumours contained a malignant component derived from muscle ; malignant connective tissue elements were also present in some of these 13, and in the remaining 4 tumours the malignant process appeared to have arisen predominantly in the connective tissue. The tumours were rhabdomyosarcomata, rhabdomyofibrosarcomata, pleomorphic and fibrosarcomata.

The purpose of the present investigation was to follow step by step and in some detail the tissue changes caused by the injection of powdered cobalt metal into rat skeletal muscle, up to the stage at which frankly malignant changes could be recognised.

### MATERIALS AND METHODS

Two experiments were made. In the first the rats were killed at fortnightly intervals ; since the results showed that pronounced changes were already present in the tissue at 2 weeks, a second experiment was undertaken in which the animals were killed at intervals of 1 to 28 days after injection.

Male rats of the hooded strain aged 2–3 months were used. Thirty animals were injected in the thigh muscle of the right leg with 0·028 g. cobalt metal powder (spectrographically pure) shaken into suspension with 0·4 ml. of fowl serum ; 15 controls were similarly injected with 0·4 ml. fowl serum only. At intervals after the injection animals taken at random were killed by cervical dislocation, and the portion of the thigh muscle surrounding the injection site was excised. Pieces of the excised muscle were fixed in Carnoy's fluid for subsequent staining with methyl green-pyronin (MGP), and in Zenker's fluid for Azan staining ; tissue fixed in Carnoy's fluid, however, also gave satisfactory results with Azan.

### RESULTS

As in the previous experiments, little or no local reaction to the injection could be detected by examination of the intact animal, and there were no general toxic effects. Study of the histological material, however, revealed a distinct and characteristic chain of events in the cobalt-treated rats but nothing of consequence in the controls injected with serum only. The progressive changes in the cobalt-treated animals led from the initial local trauma, through a stage of cell proliferation up to the malignant change and the final development of tumours of the type previously described (Heath, 1956).

There appear to be two main processes at work :—

(1) The response of the muscle to the mechanical injury produced by the injection of the metal grains, as shown by an attempt at regeneration and repair.

(2) The modification of the regenerative and repair process by the chemical action of the cobalt presumably either by slow solution in the tissue fluids to give cobalt ions, or by direct catalysis at the surface of the metal grains.

These two processes are clearly seen in the histological material and are very similar in all rats affected. In a few animals the injury heals normally, and in others which are not considered in detail here, the progressive changes leading to malignancy occur in the connective tissue alone.

The first response of the muscle to the injection appears at 1 day as an infiltration of leucocytes into the spaces between the muscle bundles and fibres, in regions near the primary injury (Fig. 1). This infiltration is still seen at 4 days but by this time many fibroblasts have appeared in the region. Large aggregates of cobalt grains can be found in immediate contact with intact muscle which sometimes shows no evidence of damage (Fig. 2), whereas at sites distant from the cobalt some muscle fibres contain a greatly increased population of nuclei (Fig. 3). A nucleosis of this type has been described by Altschul (1947) who attributes it to the loss of equilibrium in pressure between the nuclei and the sarcoplasm and fibrillae consequent upon the injury. In other damaged muscle fibres both nuclei and striations have disappeared to leave a homogeneous hyaline material.

The muscle continues to degenerate and at 7 days necrotic unstriated bundles often lie between bundles of normal striated fibres (Fig. 4) ; a further breakdown of this hyaline substance into amorphous granular residues now begins. The zones between adjacent muscle fibres, particularly where one fibre is necrotic, become filled with cells of many types, leucocytes, fibroblasts and characteristic fusiform cells. These last (Fig. 5) are sometimes mononucleate and sometimes have 2–5 nuclei ; their cytoplasm is very basophilic and stains deeply with pyronin. They appear to be myoblasts, possibly derived from the injured muscle fibres by the process of " dissociative degeneration " described by previous authors (Pfühl 1937, Betz 1951, quoted by Godman 1957) ; this process is said to involve the freeing of nuclei from a muscle fibre, each with its own complement of endoplasm, to form mononucleate myoblasts. In other areas there are multinucleate cell tubes (Fig. 6) the cytoplasm of which scarcely stains with pyronin, which correspond to the cell tubes of Waldeyer (Waldeyer 1865, quoted by Godman 1957) and represent collapsed muscle fibres. Regeneration has thus begun.

Extensive areas of degeneration are still present at 12 days with increasing amounts of the amorphous granular material, but regeneration is now well under way ; in some zones many long multinucleate basophilic or striated muscle straps are seen (Fig. 7) which sometimes at least are continuous with damaged muscle bundles. Waldeyer's tubes with faintly staining cytoplasm are still present. Collagen fibres have now appeared in some rats and numerous mast cells are scattered amongst them.

So far the picture of the damage due to the injury and of the subsequent attempts at repair and regeneration agree very closely with the findings of Godman (1957) in the repair of infarcted muscle ; the production of fusiform myoblasts, probably from damaged muscle fibres by " dissociative degeneration ", is common in Godman's material as in ours. Between 2 and 3 weeks after the injection of cobalt, however, the tissue response begins to differ from that described by

Godman. Fusiform myoblasts (Fig. 8) continue to be formed but they show progressively less tendency to associate and mature into differentiated fibres. Whereas in the muscle infarction undifferentiated myoblastic elements diminish in number after 16 days, in the cobalt-treated material they continue to increase.

At 4 weeks changes due to the action of the cobalt have spread further from the metal deposit to more distant regions of the muscle (Fig. 9–15). The cobalt granules which are still present are usually surrounded by a narrow band of necrotic material containing pycnotic nuclei and also some degenerate muscle (Fig. 9). Two or three cell widths away there is a broad zone of viable cells of various types but mainly leucocytes and fibroblasts (Fig. 10) beyond which are viable muscle fibres (Fig. 11). At this stage the changes in the muscle fibres are much more readily followed than in the earlier material and the histological

---

### EXPLANATION OF PLATES

FIG. 1.—Infiltration of leucocytes into the space between muscle bundles and fibres. One day after injection of cobalt. Stained Methyl-Green and Pyronin (MGP). ×450.

FIG. 2.—Grains of cobalt metal in intimate contact with apparently undamaged muscle. Four days. MGP. ×450.

FIG. 3.—Increased numbers of nuclei at a site distant from the injected cobalt but in the same muscle. Four days. MGP. ×450.

FIG. 4.—Damaged muscle fibre showing its hyaline, almost homogeneous nature, in contrast with the neighbouring undamaged fibre. Seven days. MGP. ×450.

FIG. 5.—Fusiform cell with very basophilic cytoplasm staining deeply with pyronin and having possibly 5 nuclei. Seven days. MGP. ×450.

FIG. 6.—Multinucleate cell tubes in which the cytoplasm scarcely stains at all with pyronin; these are the cell tubes of Waldeyer. Seven days. MGP. ×450.

FIG. 7.—Region of regeneration containing numerous multinucléate cells of both types, some with cross striations. Twelve days. MGP. ×200.

FIG. 8.—Free myoblast, possibly binucleate, with cytoplasm staining deeply with pyronin. Nineteen days. MGP. ×450.

FIG. 9.—Cobalt granules still present surrounded by a narrow band of necrotic material containing pycnotic nuclei and also some granular degenerate muscle substance. Four weeks. MGP. ×950.

FIG. 10.—Very cellular zone of considerable width lying between cobalt grains and intact muscle fibres. Four weeks. MGP. ×950.

FIG. 11.—Abutment of the cellular zone upon the intact muscle fibres. Four weeks. MGP. ×950.

FIG. 12.—Abnormal fibres with strings of peripherally situated nuclei. Four weeks. MGP. ×950.

FIG. 13.—Damaged muscle fibre with a collection of peripheral nuclei wrinkling up the sarcolemma. Four weeks. Azan. ×950.

FIG. 14.—Damaged muscle fibre with a chain of nuclei disturbing the regular pattern of striation. The nuclei and nucleoli are larger than normal and stain much more deeply. Four weeks. Azan. ×950.

FIG. 15.—Nuclei in affected muscle showing polar caps of pyronin staining endoplasm. Four weeks. MGP. ×950.

FIG. 16.—Two myoblasts in a region containing much collagen. Note the densely staining nucleoli. Six weeks. MGP. ×950.

FIG. 17.—Binucleate myoblasts, and a mitosis. Ten weeks. MGP. ×950.

FIG. 18.—Giant cell similar to those found in the established cobalt-induced tumours. Fourteen weeks. MGP. ×950.

FIG. 19.—Two myoblasts showing cross striations. Fourteen weeks. MGP. ×950.

FIG. 20.—Attempt at production of differentiated muscle fibre in the transplanted cobalt-induced tumour (32nd passage). MGP. ×450.

FIG. 21.—Mitosis in a region with much necrotic material in an early tumour nodule. Twenty weeks. MGP. ×950.

FIG. 22.—Degenerating muscle substance: granular pigmented deposits staining from reddish-brown to yellow and green. Sixteen weeks. MGP. ×950.

FIG. 23.—Mitosis in giant cells in the same nodule as shown in Fig. 21. Twenty weeks. MGP. ×950.

Heath,

Heath.

Heath.

14

15

16

17

18

Heath.

**Heath.**

picture lends support to the view that the mononucleate myoblasts are derived from mature fibres by the dissociative degeneration mentioned above. In the cobalt-treated material muscle fibres closest to the cobalt implant differ from normal fibres in the presence of strings of nuclei along the periphery (Fig. 12) ; the nuclei may be so close to the surface of the cytoplasm that the sarcolemma is wrinkled and the striations disturbed giving the appearance of an outpouring of nuclei from the interior of the muscle fibre (Fig. 13 and 14). In some areas both the nuclei and nucleoli in these strings are larger and more deeply staining than normal (Fig. 14) ; other nuclei are associated with large polar caps of basophilic pyronin-staining material (Fig. 15) the so-called endoplasm referred to above, which according to Altschul (1947) is the true cytoplasm of the muscle cells and independent of the sarcoplasm and fibrillae of the muscle-fibre proper. This " dedifferentiation " of mature muscle fibres continues under the action of cobalt whereas an isolated mechanical trauma only evokes a limited degree of dissociation sufficient to repair the damage (Godman, 1957). Free myoblasts appear in the zones near the cobalt injection ; at 6 weeks these cells are larger than in the initial stages of the tissue response, assume more and more abnormal forms, and usually have very basophilic cytoplasm and large deeply staining nucleoli. They may be present between existing muscle bundles, in the residual necrotic material, and in masses of collagen (Fig. 16).

At 8 to 10 weeks free myoblasts are very abundant and many are binucleate. Mitoses are now seen but whether in myoblasts or other cell types has not been established (Fig. 17). At 14 weeks giant cells (Fig. 18) appear in increasing numbers and although their origin is not certain, similar cells are common in the fully developed cobalt-induced tumour. Myoblasts abound, and while some show cross-striations (Fig. 19) most do not.

The continuing action of cobalt on the newly formed myoblasts probably prevents them from redifferentiating into striated elements as they do in repair after infarction. That some capacity for redifferentiation may persist at least in some cells is suggested by the fact that multinucleate straps occasionally appear even in cobalt-induced tumours that have been transplanted for many generations (Fig. 20).

At 16 weeks muscle substance continues to degenerate into pigmented granular masses (Fig. 22) and mitoses are now very frequent, in spite of the fact that cobalt is still present ; they even occur in the largely necrotic regions adjacent to metal grains. In one rat cobalt is still present at 20 weeks and a tumour nodule is just discernible (Fig. 21, 23).

### DISCUSSION

The study of the histogenesis of these cobalt-induced tumours has revealed that under the experimental conditions described, cobalt causes an extensive and continuing reversal of the normal processes whereby embryonic mononucleate myoblasts associate to form fully differentiated muscle fibres (Firket, 1957). It seems clear that it is from myoblasts of this type, probably originating through the breakdown of mature fibres, that the malignant variants arise and that these in turn develop into the established tumour.

The process is of interest partly because the breakdown of such a highly differentiated tissue is very easy to follow visually, and partly because the production of such tumours from muscle by experimental carcinogens is either uncommon or has not been often reported. The fact that the carcinogen in these studies is an element, the metal cobalt, and therefore can be followed throughout the organism by various chemical and tracer techniques will enable the investigation to be pursued further in several directions, and gives some grounds for hope that the nature of one particular carcinogenic mechanism can be found.

The work of several authors has shown that cobalt can inhibit the respiration of various tissue and cells. Unpublished experiments by my colleagues, Dr. M. Webb, Mr. J. T. Dingle, Dr. M. R. Daniel and myself, now in progress in this Laboratory, show that cobalt is a poison for some respiratory systems of rat tissues. Experiments are being made to find whether there is a correlation between the dedifferentiation of muscle tissue described here and the inhibition of respiration, and if so what is the nature of the connection.

### SUMMARY

The injection of cobalt metal powder into the thigh muscle of rats regularly produces a high incidence of characteristic malignant tumours, many of which are derived from the muscle tissue itself. The steps in this process have been followed and found to be very similar in different rats. The carcinogenic process appears to be firstly an extensive and continuing breakdown of the differentiated muscle fibres into free myoblasts, and secondly the transformation of some of these myoblasts into malignant variants. A possible mechanism is suggested.

The author is deeply indebted to his technical assistants, Mrs. Audrey Thompson and Miss Angela Orledge for their skill and patient attention to detail without which this work would not have been possible. He also wishes to thank Dr. Honor B. Fell, F.R.S. and his other colleagues for the many helpful and stimulating discussions which have played their part in the interpretation of these findings.

The work was financed by the British Empire Cancer Campaign.

### REFERENCES

ALTSCHUL, R.—(1947) *Rev. canad. Biol.*, **6**, 485.
BETZ, H.—(1951) *Arch. Anat. micr. Morph. exp.*, **40**, 46 (quoted by Godman).
FIRKET, H.—(1957) Thesis. Faculté de Médecine. Université de Liège. ‘ Recherches sur la synthèse des acides désoxyribonucléiques et la préparation à la mitose dans des cellules cultivées *in vitro* ’.
GODMAN, G. C.—(1957) *J. Morph.*, **100**, 27.
HEATH, J. C.—(1956) *Brit. J. Cancer*, **10**, 668.
PFÜHL, W. (1937) *Z. mikr.-anat. Forsch.*, **41**, 569 (quoted by Godman).
WALDEYER, W.—(1865) *Virchows Arch.*, **34**, 473 (quoted by Godman).